# Border Trap Extraction with Capacitance- Equivalent Thickness to Reflect the Quantum Mechanical Effect on Atomic Layer Deposition High-*k*/In_0.53_Ga_0.47_As on 300-mm Si Substrate

**DOI:** 10.1038/s41598-019-46317-2

**Published:** 2019-07-08

**Authors:** Md. Mamunur Rahman, Jun-Gyu Kim, Dae-Hyun Kim, Tae-Woo Kim

**Affiliations:** 1 School of Electrical Engineering, University of Ulsan, Ulsan, 44610 Korea; 2 School of Electronics Engineering, Kyungpook National University, Daegu, 702-701 Korea

**Keywords:** Electrical and electronic engineering, Electronic devices

## Abstract

This study presents a model to calculate the border trap density (N_bt_) of atomic layer deposition high-*k* onto In_0.53_Ga_0.47_As on a 300-mm (001) Si substrate. This model considers the quantum confinement effect and band nonparabolicity. Capacitance-equivalent thickness (CET) was used to reflect the distance of the charge centroid from the oxide–semiconductor interface. The border trap values based on CET were found to be approximately 65% lower than the extracted values based on physical thickness in the In_0.53_Ga_0.47_As material. In an investigation of two different post-metal annealing effects on border traps, the border trap was more effectively passivated by N_2_-based forming gas annealing (FGA) compared with rapid thermal annealing (RTA), whereas a lower interface state density was observed in RTA-annealed samples compared with FGA-annealed samples. N_bt_ extraction at different bias voltages demonstrated that the applied frequencies travel deep into the oxide and interact with more traps as more the Fermi level passes the conduction band, thus creating tunneling with the carriers.

## Introduction

As a substitute for conventional SiO_2_/n-type Si metal-oxide-semiconductor field-effect-transistors (MOSFETs), the combination of different higher dielectric constant (*k*) oxides along with In_x_Ga_1−x_As (*x* > 0.5) as a channel material has been extensively investigated for its potential equivalent oxide thickness (EOT) scaling and use in forthcoming logic-applicable devices that will require high-speed and depressed power consumption^1–5^. Among the different high dielectric constant (*k*) oxides, studies on Al_2_O_3_, HfO_2_, La_2_O_3_, ZrO_2_, and their nanolaminate and nano-mixture structures have already been reported^6–11^.

In_0.53_Ga_0.47_As is often used instead of silicon because of its high electron mobility, which results from its lower effective mass of electrons. However, because of this lower effective mass of electrons, In_0.53_Ga_0.47_As suffers from low density of states (DOS)^12^. To be more precise, the DOS of In_0.53_Ga_0.47_As is less than that of Si by approximately two order of magnitudes^13,14^. As a result, this insufficient DOS causes the Fermi level of In_0.53_Ga_0.47_As to move inside the conduction band; otherwise, the benefit of its high mobility would be lost^4^. The band offset of high-*k* and In_0.53_Ga_0.47_As substrate is comparatively smaller than that of a conventional SiO_2_/Si interface.

Once the fermi level, *E*_*F*_, has biased near the conduction band, the effective barrier height between the oxide and In_0.53_Ga_0.47_As, which is denoted as *E*_*ox*_ − *E*_*F*_, is reduced. As a result, the trap inside the oxide starts the charge/discharge process with electrons from the semiconductor through tunneling^14^. These traps, which are positioned in the dielectric near the oxide–In_0.53_Ga_0.47_As interface, are commonly known as *border traps*^15–17^. The idea of these traps was first introduced by Fleetwood^15^. Border traps are characterized by their location: the farther they are from the interface, the longer it takes for the majority carrier to fill them. Their charge exchange is also determined by the applied alternating current (ac) frequency. A lower frequency provides deep tunneling by decreasing the apparent thickness of the oxide, whereas the tunneling probability decreases at a higher frequency^16^.

**Figure 1:**
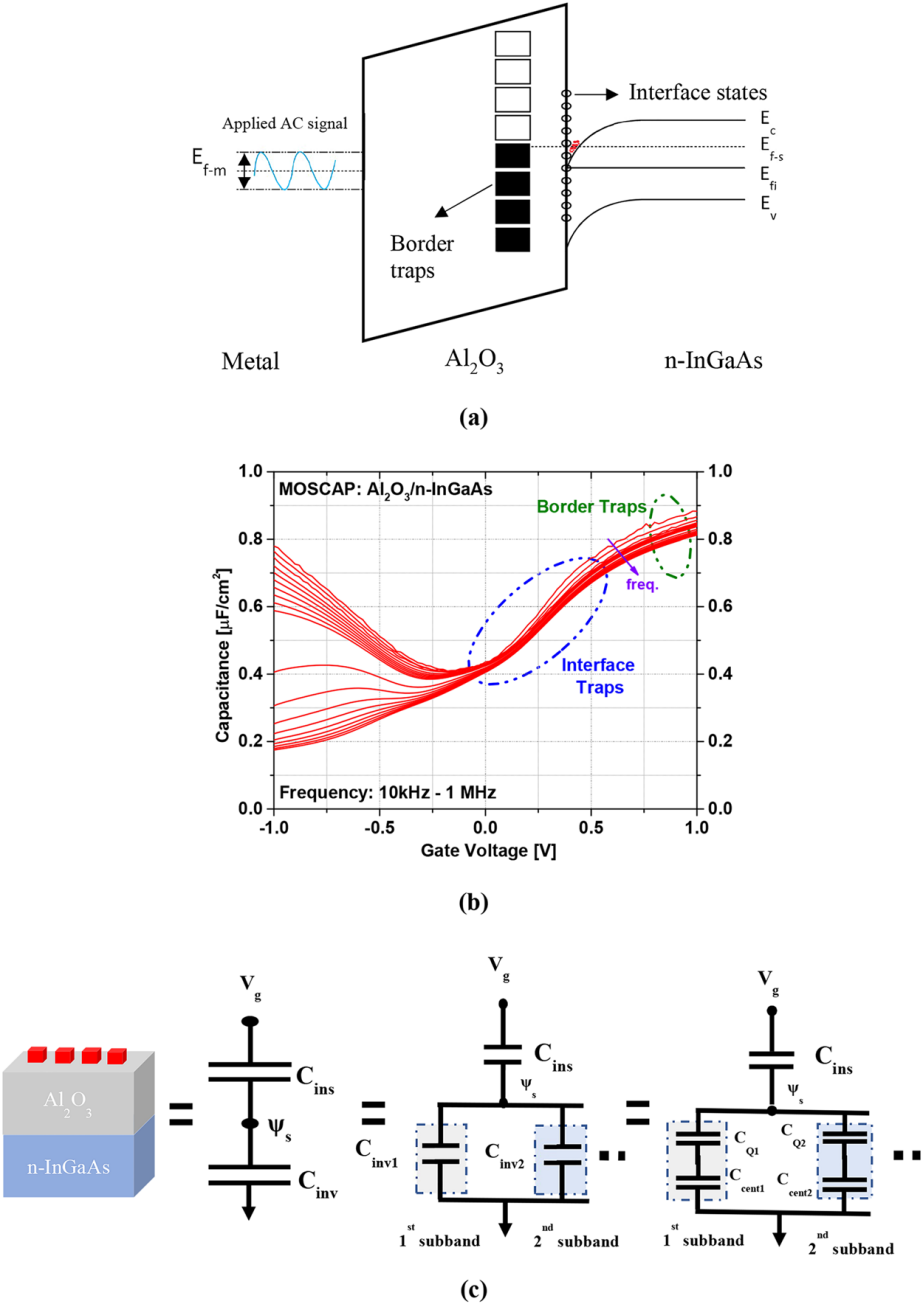
(**a**) Energy-band diagram of a metal/Al_2_O_3_/n-InGaAs MOS capacitor with the interface and border traps when an AC signal is applied. (**b**) Response regions of the interface and border traps in the capacitance-voltage (C-V) behavior of the Al_2_O_3_/n-InGaAs MOS capacitor. (**c**) Equivalent circuit diagram for the gate capacitance of the III–V MOSFET.

Figure 1a shows how the tunneling of electrons occurs from the semiconductor to oxide layer in response to an applied AC frequency with sufficient direct current (DC) bias voltage. However, the effect of these near-interfacial dielectric traps is a great stimulus for the on-state act of a MOSFET. Because the fermi level (*E*_*F*_) is pinned inside the conduction band, border traps prevent the formation of sufficient carriers in the channel, which leads to reduced carrier mobility by phonon scattering and eccentricity of the threshold voltage^17,18^. These traps are also responsible for reduction of gate voltage control on the channel current, enhancing gate leakage current, a degradation of transconductance as well as for hysteresis^19,20^. The impact of border traps is more prominent in the accumulation region, where a dispersion is always observed in the capacitance-voltage (C-V) response of the metal-oxide-semiconductor (MOS) capacitor because of the transportable carrier exchange among the border traps and conduction band states via tunneling, as described previously^21–24^.

Figure 1b illustrates the region where border traps are prominent as an active trap state in capacitance-voltage (C-V) with a frequency dependency from 10 kHz to 1 MHz. Unlikely, the typical interface state model cannot explain this dispersion because the time constant of these traps at accumulation is much smaller than that of the bulk oxide traps, within the typical measurement frequency range from 1 kHz to 1 MHz^25^. The electrical behavior of border traps is quite different from that of interface traps in several ways. Firstly, the interface traps are inactive at the energy value of the accumulation region, where the frequency scattering occurs^22^. Secondly, compared with the time constant that is responsible for the interface trap’s charging/discharging, the dispersion performance is less temperature dependent because of the border traps^26^. Finally, the chemical treatment has no effect on border traps but successfully diminishes the dispersion caused by interface traps^27^.

Researchers have proposed several methods for modelling the dispersion of capacitance at accumulation and making electrical characterizations of border traps^22–25,28–31^. Among them, the distributed border trap model from Yuan *et al*. is the most well known^28,31^. In this model, the oxide thickness (*t*_*ox*_) is segregated into immeasurable quantities of small fragments, with each part contributing an amount of oxide capacitance, Δ*C*_*ox*_, connected in parallel with the admittance proportional to the border trap density; this parallel combination is connected in series with the semiconductor capacitance. The border trap density is then extracted by making a best-fit condition between the calculated capacitance and the conductance achieved from this model and experimental values. Usually, this oxide thickness is mainly the physical thickness (*t*_*ox*_) or EOT, which is the result of either ellipsometry or transmission electron microscopy analysis.

When determining the semiconductor capacitance *C*_*s*_, it is not clear that previous studies’ consideration about the quantum mechanical effect^12,25,32–34^. For practical purposes, this capacitance is not only the oxide capacitance but also a series combination of inversion capacitance (*C*_*inv*_), which includes quantum capacitance (*C*_*Q*_) and centroid capacitance (*C*_*cent*_), by assuming that the first electron sub-band in the channel is occupied^35,36^. Therefore, the total capacitance includes a series combination of insulator capacitance (*C*_*ins*_), quantum capacitance (*C*_*Q*_), and centroid capacitance (*C*_*cent*_). The equivalent electrical circuit is shown in Fig. 1c. In a large-scale device, the series combination of *C*_*Q*_ and *C*_*cent*_ is larger than *C*_*ox*_, so the gate capacitance approaches only *C*_*ox*_. However, in small-scale devices, the oxide thickness is on a nanometer scale; thus, *C*_*ox*_ becomes commensurate with the other components of total capacitance^35^.

Quantum capacitance (*C*_*Q*_), which is proportional to the DOS of the channel material, physically originates from the Fermi-level penetration onto the conduction band. In the III–V channel material, a two-dimensional electron gas requires energy to be created in the semiconductor quantum well region because of low density-of-states (DOS). Therefore, the Fermi level moves above the conduction band to increase the charge in the quantum well. This movement requires energy and conceptually is equivalent to quantum capacitance. However, charges in the quantum well take a bell-shaped distribution rather than distributing themselves in a sheet form with zero thickness, which means that the physical distance of each charge is quite different from the metal gate. Moreover, the center of the charge distribution may be away from the insulator–semiconductor interface due to the confinement in the quantum well. These effects should be considered when modelling the total capacitance. When these factors are excluded, the overall capacitance is overestimated, so the extracted border trap density (*N*_*bt*_) is inaccurate^32^. To improve the extraction of border trap density, total semiconductor capacitance (*C*_*s*_) should be included with the quantum confinement effect and band nonparabolicity in the model. Additionally, because of the quantum mechanical confinement consequence of the carriers, the supplementary thickness of the conductive channel should be considered because the charge centroid in the conductive channel is located deeper beneath the interface of the dielectric and semiconductor, as described previously^37^.

Capacitance-equivalent thickness (CET) reflects a more realistic set-up because it considers the above-mentioned effect. Therefore, border trap extraction using a CET metric is a more reliable way to obtain the accurate density of border traps. In this study, we used the CET metric for the extraction of *N*_*bt*_ in addition to using physical thickness and determined that border trap density is overestimated when the quantum mechanical effect is not considered. In addition, because a border trap is an oxide’s native property, the parameters of oxide growth from atomic layer deposition (ALD) must have some effects on border trap formation. In this regard, we also examined the effects of ALD growth temperature on border traps. Moreover, several studies have passivated border traps using a variety of annealing processes^33,38^. Thus, we also examined the passivating effects of two types of annealing processes on border traps. Lastly, the interface trap density (D_it_) was also extracted for samples that were differentiated with the passivation scheme.

## Model Description

### Gate capacitance model

The total capacitance of III–V MOS structures is modelled as a series combination of insulator capacitance (C_ins_) and inversion capacitance (C_inv_) by assuming no doping level underneath the channel as demonstrated in Fig. 1c. It consists of a parallel combination of contributions of each occupied electron subband in the channel. From the figure it is evident that, for each subband *i*, the inversion-layer capacitance (C_inv_i_) consists of the quantum capacitance (C_Q_i_) and the centroid capacitance (C_cent_i_) which are connected in series. So, inversion-layer capacitance can be defined as

1 $${C}_{inv\_i}=\frac{\partial (\,-\,{Q}_{s})}{\partial {\psi }_{s}}=\frac{q\partial (\,-\,{Q}_{s})}{\partial ({E}_{F}-{E}_{C})}$$

where, *ψ*_*s*_ is the surface potential, E_F_ is the fermi level, E_C_ is the conduction band edge at the barrier-channel interface on the channel side, and Q_s_ is the total electron charge in the channel which is the sum of all the charges in each of the sub bands. This can be formulated as

2 $${Q}_{s}=\sum _{i}\,{Q}_{i}=\sum _{i}\,{\int }_{{E}_{i}}^{\infty }\,\frac{\frac{{m}_{||}^{\ast }q}{\pi {\hslash }^{2}}}{1+exp\,(\frac{E-{E}_{F}}{kT})}dE$$

where, Q_i_ is the electron charge of subband *i* in the channel, E_i_ is the energy level of subband *i*, and ${{\rm{m}}}_{||}^{\ast }$ is the in plane effective mass of the channel material. The effective mass is a function of energy due to nonparabolicity of the band structure which can be expressed as follows^39^.

3 $${m}_{\parallel }^{\ast }={m}_{0}^{\ast }(1+\alpha E)=\frac{{\hslash }^{2}{k}^{2}}{2E}$$

where, ${{\rm{m}}}_{0}^{\ast }$ is the effective mass at k = 0, E and k are the energy and wave number of the charge carrier, ħ is the reduced Plank’s constant and α is the nonparabolicity parameter.

Now, we can define quantum capacitance for any subband (C_Q_i_) as the derivative of electron charge in subband *i* with respect to energy difference between E_F_ and E_i_. Mathematically,

4 $${C}_{Q\_i}=\frac{q\partial ({Q}_{i})}{\partial ({E}_{F}-{E}_{i})}=\frac{q\partial (-\,{\int }_{{E}_{i}}^{\infty }\,\frac{\frac{{m}_{||}^{\ast }q}{\pi {\hslash }^{2}}}{1+exp\,(\frac{E-{E}_{F}}{kT})}dE)}{\partial ({E}_{F}-{E}_{i})}=\frac{\frac{{m}_{||}^{\ast }{q}^{2}}{\pi {\hslash }^{2}}}{1+exp\,(\frac{{E}_{i}-{E}_{F}}{kT})}.$$

Similarly, centroid capacitance of subband *i* (C_cent_i_) can be defined as the derivative of electron charge in subband *i* with respect to energy difference between E_i_ and E_C_.

5 $${C}_{cent\_i}=\frac{q\partial (\,-\,{Q}_{i})}{\partial ({E}_{i}-{E}_{C})}={C}_{Q\_i}.\frac{\partial ({E}_{F}-{E}_{i})}{\partial ({E}_{i}-{E}_{C})}$$

Then C_inv_i_ can be formulated as

6 $${C}_{inv\_i}=\sum _{i}\,{(\frac{1}{{C}_{Q\_i}}+\frac{1}{{C}_{cent\_i}})}^{-1}$$

So, if the location of each subband energy level (E_i_) and the fermi level (E_F_) are known with respect to conduction band edge, then all the capacitance component can be evaluated.

### Border trap extraction model

**Figure 2:**
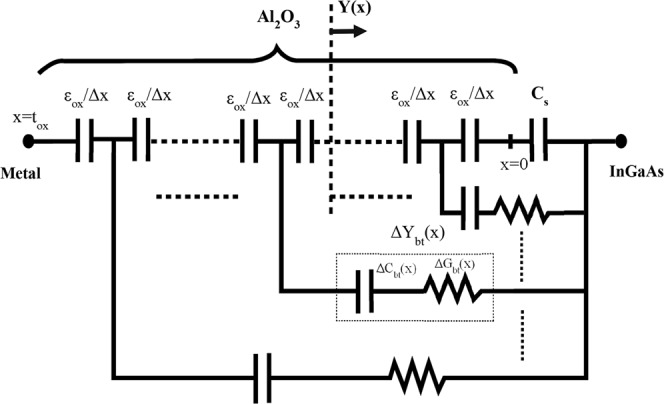
Equivalent electrical circuit representation of an MOS capacitor using the distributed bulk oxide trap model^28,31^.

For a quantitative analysis of border trap density, we followed the distributed border trap model as mentioned previously. This model enumerates the border traps by analyzing the frequency dispersion at an accumulation region at any specific bias voltages. The whole oxide capacitance is modelled onto an abundant number of small capacitive components, Δ*C*_*ox*_, where each component characterizes the capacitance of a very small piece of oxide thickness. The induced charge storage and energy loss from the border traps are modelled by a series of connected capacitance (Δ*C*_*bt*_) and conductance (Δ*G*_*bt*_) for any portion of oxide thickness. This admittance due to border traps is connected in parallel to the oxide capacitance. This parallel combination is then connected in series with the semiconductor capacitance. Figure 2 shows the equivalent electrical circuit of the model. The whole model can be described by a first-order non-linear ordinary differential equation, as follows:

7 $$\frac{dY}{dx}=-\,\frac{{Y}^{2}}{j{\rm{\omega }}{\varepsilon }_{{\rm{ox}}}}+\,\frac{{q}^{2}{N}_{bt}\,\mathrm{ln}\,(1+j\omega \tau )}{\tau }$$

with the boundary condition at *x* = 0, *Y* = *jωC*_*s*_ where Y is the total admittance at any distance *x* from the oxide–semiconductor interface; *ω* = 2π*f* is the angular ac frequency, where *f* is the measurement frequency and *C*_*s*_ is the semiconductor capacitance corresponding to a specific surface potential *ψ*_*s*_.

The other important parameters of this model are described as follows: *q* is the elementary electron charge in Coulombs; *ε*_*ox*_ = *ε*_*r*_ · *ε*_0_ is the permittivity of any dielectric film, where *ε*_*r*_ is the relative permittivity of the oxide layer and *ε*_0_ is the permittivity of the air; *C*_*ox*_ is the oxide capacitance per cm^2^, which is defined as *ε*_*ox*_/*t*_*ox*_, where *t*_*ox*_ is the thickness of the oxide layer in centimeters; *N*_*bt*_ is the volume concentration of border traps in the oxide at a distance *x* from the oxide–semiconductor interface at any energy level, expressed as eV^−1^ cm^−3^; and *τ* is the average time in seconds for electron capturing of an empty trap. *τ* maintains an exponential relationship with the distance *x*, which is the distance of the trap within the oxide from the interface. τ can be described as follows:

8 $${\rm{\tau }}={{\rm{\tau }}}_{{\rm{o}}}{e}^{2kx}\,with\,k=\frac{\sqrt{2m\ast {{\rm{E}}}_{{\rm{b}}}}}{{\rm{\hslash }}}\,$$

Here, *τ*_0_ is the capture/emission time constant of the trap having the same energy level of *τ*. *k* is the attenuation coefficient of the tunneling process, which can be described by the effective mass of dielectric film (m*), the barrier height between the conduction band of oxide and semiconductor (*E*_*b*_), and the reduced Plank’s constant (ħ). Moreover, *τ*_0_ can be described more specifically as:

9 $${{\rm{\tau }}}_{{\rm{o}}}={({{\rm{n}}}_{{\rm{s}}}{{\rm{v}}}_{\mathrm{th}}{\rm{\sigma }})}^{-1}$$

Here, *v*_*th*_ is the thermal velocity of any electron at any temperature *T*, *n*_*s*_ is the semiconductor surface’s electron density, and *σ* is the border trap’s capture cross-sectional area. As described elsewhere^13^, at the accumulation region where the Fermi level is near the conduction band, a good approximation is to consider *n*_*s*_ as equal to the DOS (*N*_*c*_) of the conduction band. Moreover, the border traps that are deeply inside of the oxide do not respond at any applied frequency *ω* having a time constant *τ* that is greater than 1/*ω*; however, the traps closer to the interface with a smaller *τ* are more willing to respond. Using the condition *ωτ* = 1, the probing depth at any applied frequency for a border trap can be calculated as follows:

10 $${{\rm{X}}}_{{\rm{p}}}\,=\frac{1}{2k}\,\mathrm{ln}\,\frac{1}{2\pi f{{\rm{\tau }}}_{{\rm{o}}}}$$

Here, *f* is the measurement frequency.

## Results and Discussion

**Figure 3:**
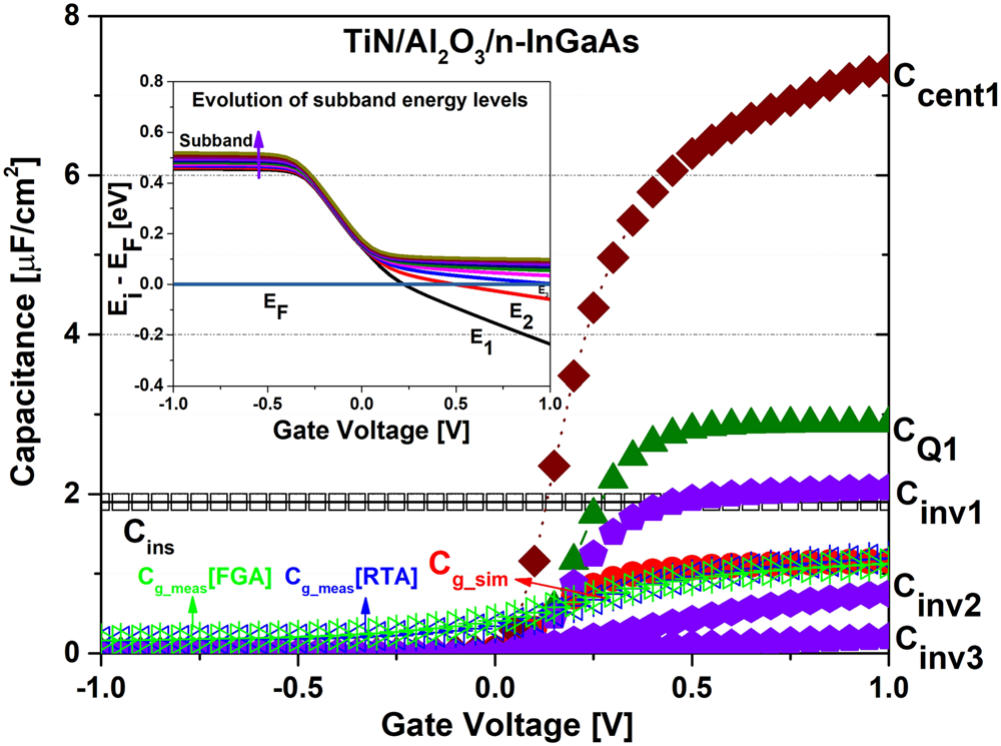
Evolution of sub-band energy levels with respect to Fermi level (inset), experimental *C*_*g*_, and modeled *C*_*g*_ components of an ALD-processed sample.

Figure 3 shows the evolution of the sub-band energy levels and all capacitance component modelling, along with experimental data from an Al_2_O_3_ ALD-deposited sample. For our modelling, we solved the self-consistent solution of the one-dimensional Poisson and Schrodinger equations using the Nextnano simulation tool^40^. This tool provides the values of sub-band energy (*E*_*i*_) and conduction band energy (*E*_*C*_) with respect to the fermi level energy (*E*_*F*_). The inset of Fig. 3 shows the evolution of the sub-band energy levels (*E*_*i*_) with respect to the fermi level (*E*_*F*_) at the applied bias voltage range. From the figure, it is evident that the fermi level penetrates the first and second energy levels while being very close to the third energy level. Thus, both the first and second sub-bands of the quantum well are populated by electrons in the operational voltage range.

Figure 3 also illustrates the measured capacitance of rapid thermal annealing (RTA) and forming gas annealing (FGA)-processed samples with modelled gate capacitance and its components. The model extraction was done by considering the conduction band effective mass of In_.53_Ga_.47_As to be 0.043m_0_ (m_0_ = electron mass at rest) by considering a non-parabolicity effect as reported in literature^41^ as well as optimizing other material parameters according to their physical structures^13^. The extracted gate capacitances were well harmonized with the modelled data. The insulator capacitance was calculated by considering the ideal dielectric constant (*k*), which is 9, and the measured oxide thickness. However, in the experimental cases, the gate capacitance approached approximately 63% of *C*_*ins*_ for the RTA-processed sample. The same condition is also valid for FGA processed samples as depicted in the Fig. 3. This capacitance deprivation mostly results from the effects of inversion capacitance. As demonstrated, *C*_*inv1*_ is quite comparable with the insulator capacitance; in addition, *C*_*Q1*_ is more dominant than *C*_*cent1*_ because of the smaller effective mass of the In_0.53_Ga_0.47_As channel. It is also clear that the capacitance dominance of any sub-band energy depends on its electron population density. Therefore, *C*_*inv2*_ is much smaller than *C*_*inv1*_ and *C*_*inv3*_ is negligible according to the fermi level penetration into the sub-band, as shown in the inset of Fig. 3.

For the extraction of border traps, the parameters of “Eq. (7)” were executed as follows. The oxide capacitance was calculated by dividing the oxide permittivity by the oxide thickness. The oxide relative permittivity was calculated according to the formula in “Eq. (11)” for every case, using the maximum capacitance value at a frequency of 10 kHz from the measured C-V data. To calculate the attenuation coefficient, the electron effective mass in Al_2_O_3_ was considered to be 0.23 m_0_ based on the literature^33^, where m_0_ is the electron mass at rest; in addition, the barrier height between the oxide and semiconductor conduction band edge was calculated using the electron affinity rule. The semiconductor capacitance *C*_*s*_ was taken at the extraction voltage of the border traps using a one-dimensional Poisson-Schrodinger solver simulation tool (Nextnano) by simultaneously considering quantum confinement and non-parabolic band effects^40^. CET was calculated according to “Eq. (12)” in the Methods section using the capacitance value from 100 kHz at the border trap extraction voltage. Both *N*_*bt*_ and *τ*_0_ were used as fitting parameters to achieve the best-fit curve for capacitance from the model by solving “Eq. (7)” with the measured data. A list of parameters that were used for the modelling is presented in Table 1.

**Table 1:** Summary of parameters used for the extraction of border trap density.

Sample	RTA-Processed Samples			FGA-Processed Samples		
Parameter	200 °C	250 °C	300 °C	200 °C	250 °C	300 °C
t_ox_ [nm]	4.2006	3.867	3.5128	4.2006	3.867	3.5128
CET [nm]	2.67	2.469	2.31	2.95	2.62	2.4
ε_r_	6.93	6.71	6.26	5.87	6.24	6.07
m*	0.23	0.23	0.23	0.23	0.23	0.23
E_b_ [eV]	3.65	3.65	3.65	3.65	3.65	3.65
k [nm^−1^]	4.5	4.5	4.5	4.5	4.5	4.5
C_s_ [μF/cm^2^]	1.08	1.143	1.22	1.08	1.143	1.22
τ_0_ [s] (Using t_ox_)	1 × 10^−11^	1 × 10^−12^	1 × 10^−12^	1 × 10^−12^	1 × 10^−12^	1 × 10^−13^
τ_0_ [s] (Using CET)	3 × 10^−11^	1 × 10^−11^	1 × 10^−12^	1 × 10^−12^	1 × 10^−11^	1 × 10^−13^
N_bt_ [cm^−3^ eV^−1^] (Using t_ox_)	1.28 × 10^20^	1.1 × 10^20^	1 × 10^20^	9.6 × 10^19^	9.75 × 10^19^	7.6 × 10^19^
N_bt_ [cm^−3^ eV^−1^] (Using CET)	4 × 10^19^	4.1 × 10^19^	3.09 × 10^19^	2.98 × 10^19^	3.58 × 10^19^	2.72 × 10^19^

**Figure 4:**
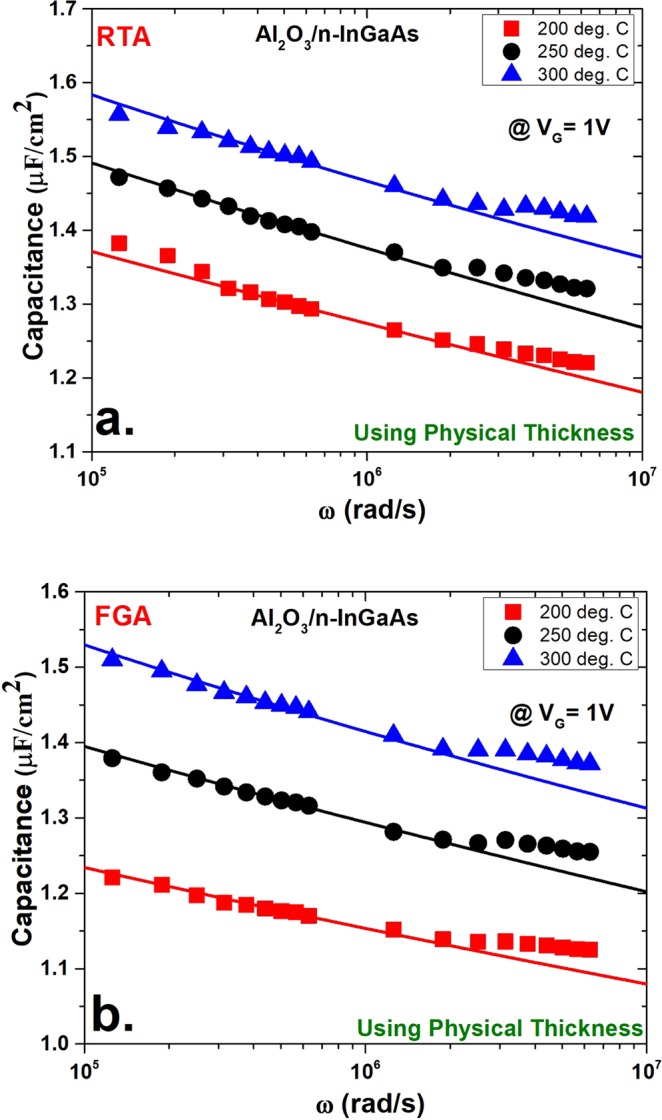
Model fitting of the capacitance versus frequency curves for the measured data (solid symbols) and calculated data from distributed border trap model (solid lines) using physical thickness (*t*_*ox*_) at an applied DC gate bias of 1 V. The fitting was performed for three different deposition conditions of Al_2_O_3_ on n-InGaAs. Shown here are the (**a**) RTA conditions and (**b**) FGA conditions.

Figure 4 shows the *N*_*b*t_ extraction fitting curves at 1 V using physical thickness *t*_*ox*_ for both the RTA- and FGA-processed cases. In both cases, the samples deposited at 200 °C show the lowest capacitance compared with the 250 °C and 300 °C deposited samples. This variation in capacitance values is mainly due to the difference in thickness between the samples deposited at different temperatures. As mentioned previously, the 200 °C processed samples showed the greatest thickness and henceforth the lowest capacitive value. The opposite case was observed for the 300 °C deposited samples. In another interesting observation, the FGA-processed samples showed somewhat lower capacitance in three different deposition cases compared with their respective RTA-processed samples. However, in the RTA-processed samples shown in Fig. 4a, there was more distortion of capacitance at the lower frequencies of the measurement window (10 kHz to 1 MHz) than at the higher ones. This disturbance in capacitance may be attributed to the noise associated with lower frequency measurements^42^. On the contrary, the FGA-processed samples did not suffer from this limitation.

In both cases at higher frequencies of the measurement window, the experimental data diverged from the fitting curve and displayed a different slope of capacitance versus log(*ω*). This change in the gradient of the capacitance versus log(*ω*) at higher frequencies is consistent at the measurement frequency range, which may be attributed to the distribution of border traps. A high concentration of traps (in the range of 10^21^ cm^−3^ eV^−1^) was reported at <1 nm from the oxide–semiconductor interface. Notably, this high density of traps that are positioned at <1 nm from the Al_2_O_3_/InGaAs interface are also found at the lower frequencies of measurement^33^. However, the extracted border trap densities of the 200 °C, 250 °C, and 300 °C deposited RTA samples were 1.28 × 10^20^ cm^−3^ eV^−1^, 1.1 × 10^20^ cm^−3^ eV^−1^ and 1 × 10^20^ cm^−3^ eV^−1^, respectively. For FGA samples, the extracted border trap densities were 9.6 × 10^19^ cm^−3^ eV^−1^, 9.75 × 10^19^ cm^−3^ eV^−1^ and 7.6x × 10^19^ cm^−3^ eV^−1^, respectively. The value of τ_0_ in these extractions was in the range of 10^−12^ s, which is attributed to the lower capture cross-sectional areas of the traps.

**Figure 5:**
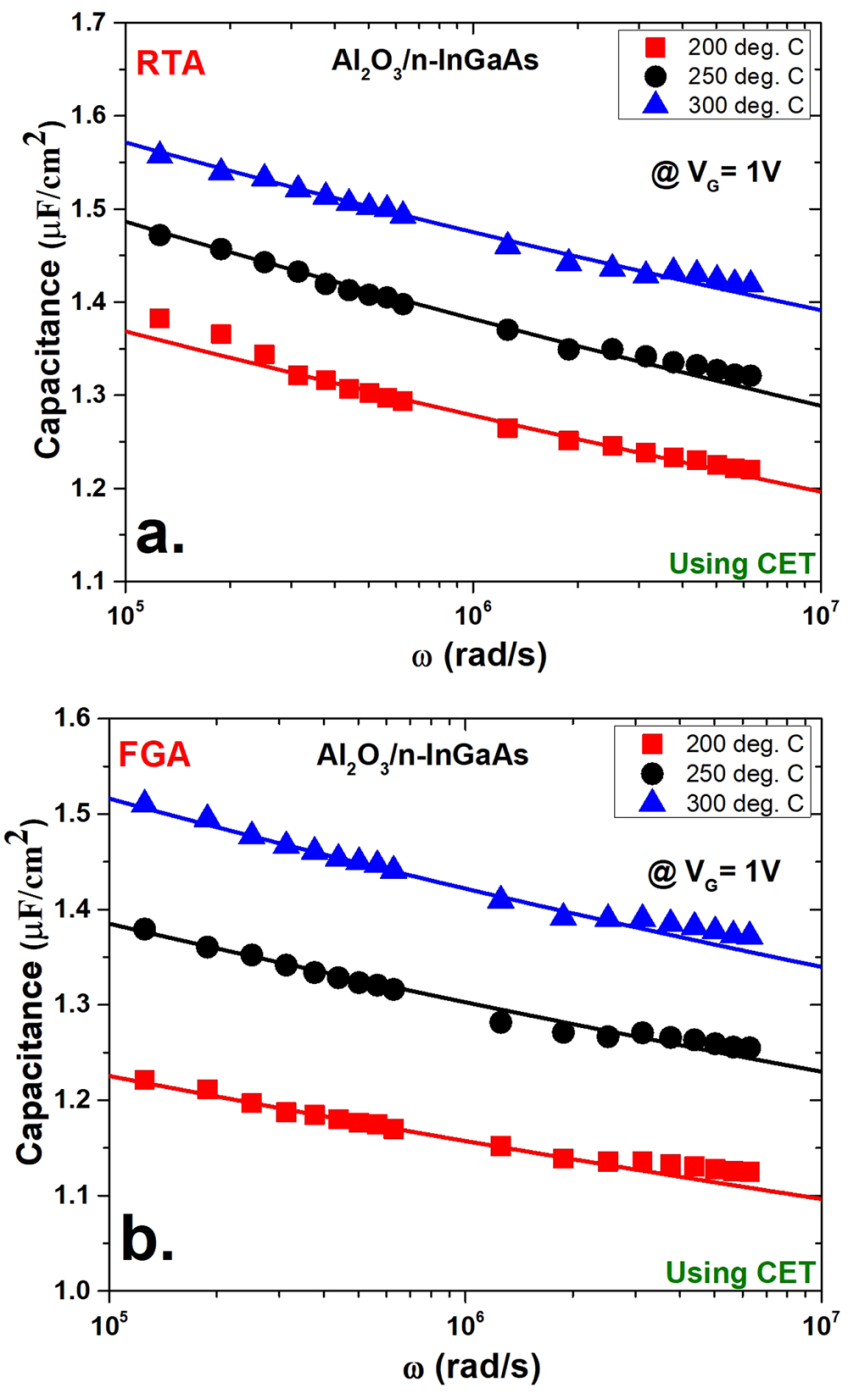
Model fitting of the capacitance versus frequency curves for the measured data (solid symbols) and calculated data (solid lines) as in Fig. 4 using capacitance-equivalent thickness (CET) at an applied DC gate bias of 1 V. Shown here are (**a**) RTA conditions and (**b**) FGA conditions.

**Figure 6:**
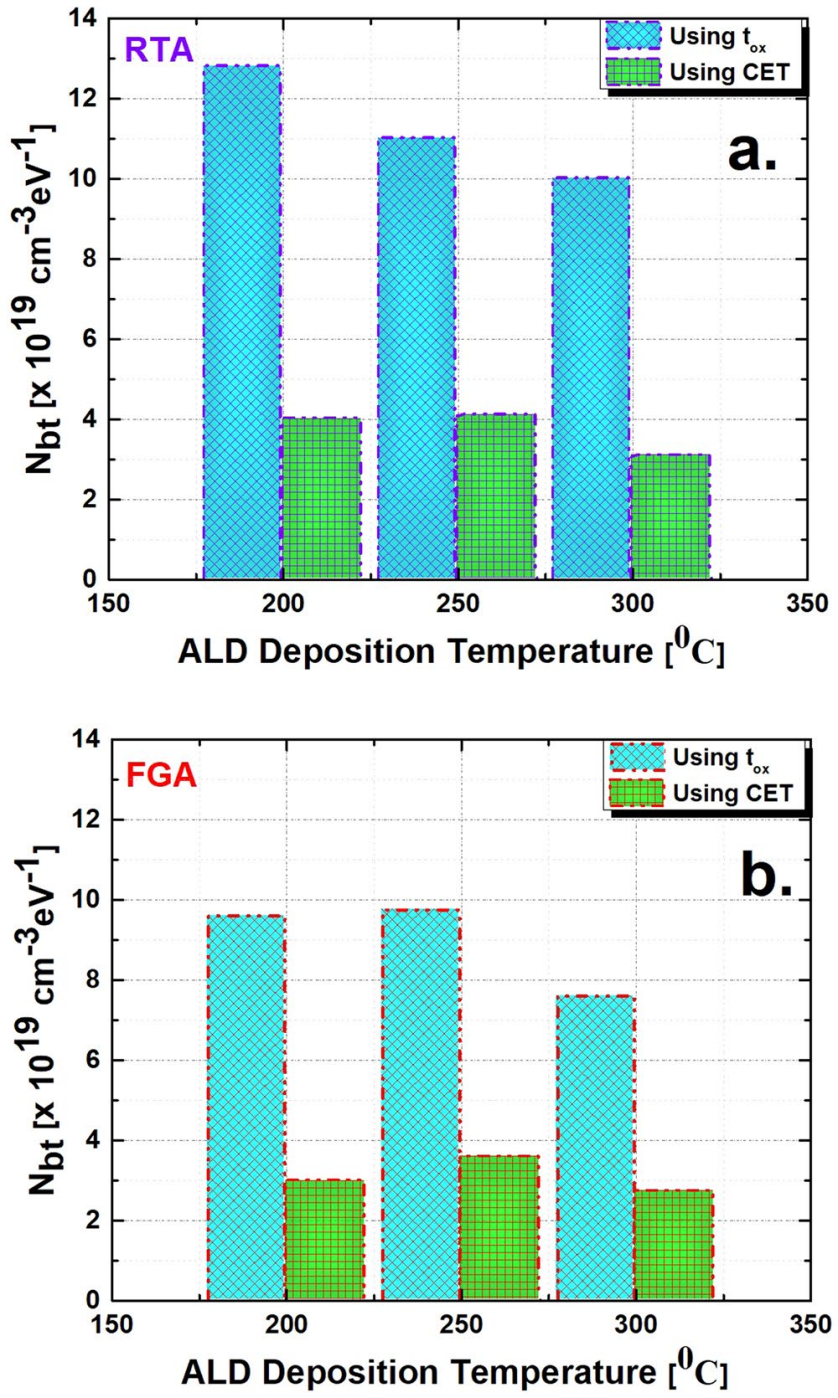
Extracted border trap density (*N*_*bt*_) difference between the physical thickness (*t*_*ox*_) and capacitance-equivalent thickness (CET)-based extraction. The comparisons were made for all three deposition conditions. Shown here are (**a**) RTA conditions and (**b**) FGA conditions.

Figure 5 shows the *N*_*bt*_ extraction for the same samples by considering the quantum mechanical effect. In this case, the physical thickness was replaced by the CET, and these CET values were extracted for all six cases. Both *N*_*bt*_ and *τ*_0_ were used as fitting parameters as before, while keeping the other parameters unchanged. Both the disturbance in capacitance at lower frequencies and the distortion of the gradient of capacitance versus log(*ω*) also existed in this case. The simulated curves obtained from the model showed a better fit than previous cases. The *N*_*bt*_ values extracted for RTA samples were 4 × 10^19^ cm^−3^ eV^−1^, 4.1 × 10^19^ cm^−3^ eV^−1^, and 3.09 × 10^19^ cm^−3^ eV^−1^ for the deposition conditions of 200 °C, 250 °C, and 300 °C, respectively. For the FGA samples, the *N*_*bt*_ values were 2.98 × 10^19^ cm^−3^ eV^−1^, 3.58 × 10^19^ cm^−3^ eV^−1^, and 2.72 × 10^19^ cm^−3^ eV^−1^, respectively. *τ*_0_ was also in the same range as earlier. However, the *N*_*bt*_ values extracted using CET were much lower than the values obtained by using physical thickness, *t*_*ox*_. This comparison is illustrated in Fig. 6a,b. The *N*_*bt*_ values by CET were approximately one third of (~65% lower than) the values by *t*_*ox*_. The overestimation of *N*_*bt*_ values using physical thickness may be because the additional thickness of the oxide layer due to quantum confinement in the In_0.53_Ga_0.47_As layer is not considered. Because the charge centroid is shifted, it causes the oxide–semiconductor interface to shift a finite thickness. As a result, the interface traps, which may contribute to energy loss, are excluded.

**Figure 7:**
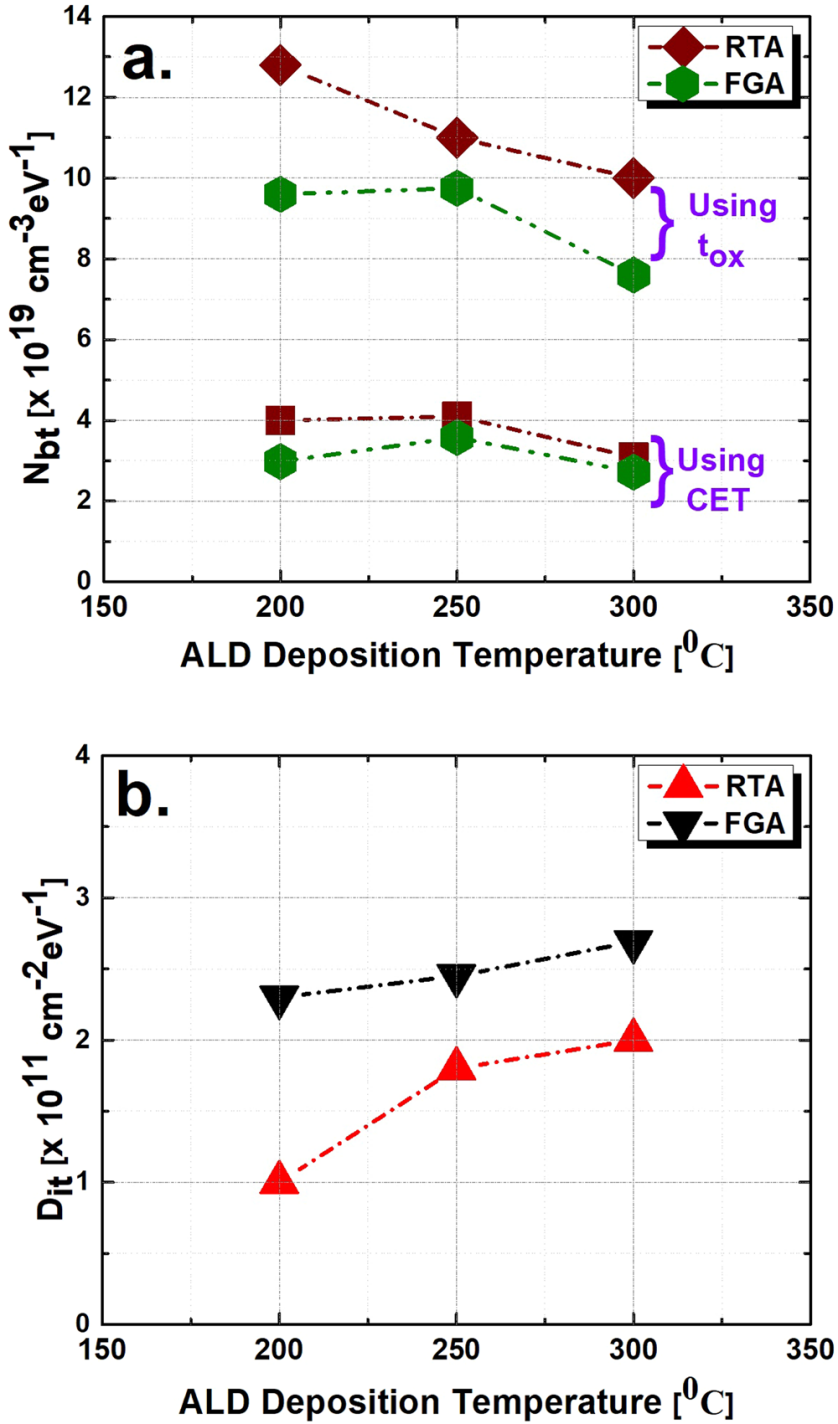
(**a**) Extracted border trap density (*N*_*bt*_) and (**b**) interface trap density (*D*_*it*_) comparisons between two different annealing processes of Al_2_O_3_/n-InGaAs samples according to their different deposition temperatures.

Figure 7a,b compare the extracted *N*_*bt*_ values of both RTA- and FGA-processed samples at three different deposition temperatures, as described previously. In both cases, the FGA-processed samples showed lower values of *N*_*bt*_ than did the RTA-treated samples. This indicates that defect passivation is more effective during the FGA process. In addition, more hydrogen (H_2_) is incorporated to passivate the dangling bonds, which is the reason for the border traps within the amorphous Al_2_O_3_ dielectric in the FGA process^38^. However, the RTA-processed samples showed comparatively lower *D*_*it*_ values than did the FGA-processed samples. Therefore, RTA treatment passivated the interface traps, whereas border traps are treated by FGA effectively. Furthermore, *N*_*bt*_ was observed to be the lowest for the 300 °C deposited sample comparing with the 200 °C and 250 °C deposited samples for both RTA- and FGA-processed cases. This lower *N*_*bt*_ indicates structural differences in the film stoichiometry at 300 °C, which leads to more hydrogen (H_2_) incorporation during FGA, as reported elsewhere^38^. Therefore, the probability of hydrogen (H_2_) bonding to defects is increased.

**Figure 8:**
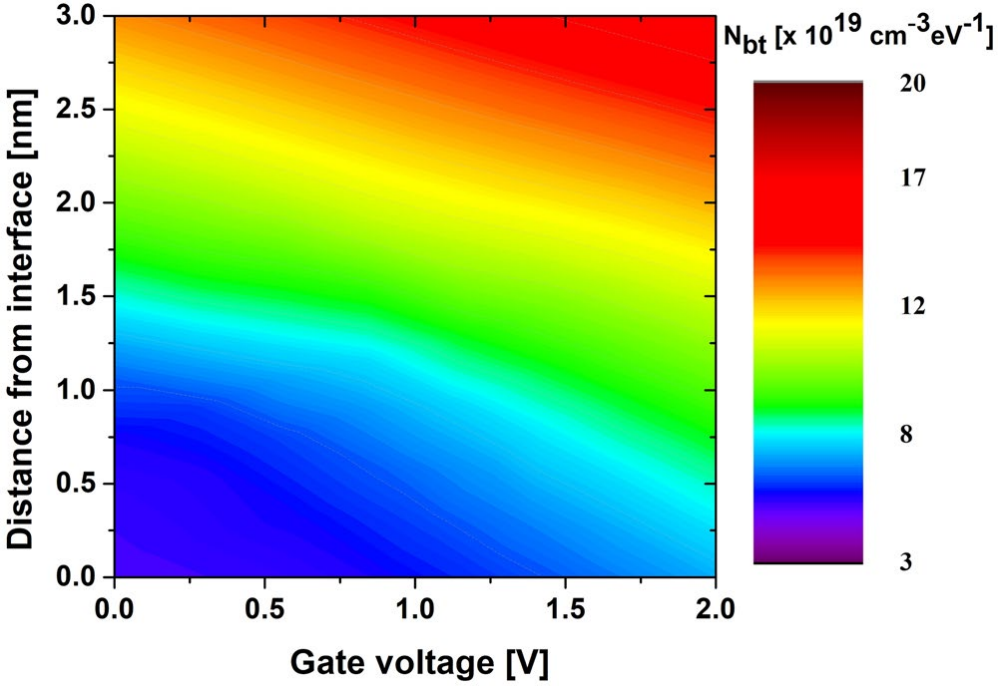
Spatial distribution of border trap density (*N*_*b*t_) as a function of applied gate voltage, as well as the location in an Al_2_O_3_ dielectric from the Al_2_O_3_/n-InGaAs interface.

Figure 8 shows the *N*_*bt*_ spatial distribution as a function of gate voltage, as well as the distance of the probing depth from the interface into the oxide. The values of *N*_*bt*_ were extracted at different applied DC voltages, whereas the values of *τ*_0_ were used to determine the probing depth into the oxide at any measurement frequency. Because border traps are more responsive at lower frequencies and the physical distance into the oxide up to where the traps respond is inversely proportional to the applied frequency, we used the lowest frequency of the measurement window (i.e., 10 kHz) to calculate the response region. The *N*_*bt*_ distribution increases as the applied voltage is increased. With the applied voltage increase, the Fermi level penetration into the conduction band also increases; in addition, the effective barrier height decreases, which makes the majority of carriers more likely to tunnel into the deep traps. When the applied voltage increases, traps with smaller electron capture cross-sectional areas in the deep oxide layer respond. Because all parameters, excluding *τ*_0_, are the same at different extraction voltages, a smaller *τ*_0_ results in a larger tunneling distance.

## Conclusion

We extracted the border trap density of an Al_2_O_3_ oxide layer deposited at three different temperatures to n-In_0.53_Ga_0.47_As on a 300-mm Si substrate by considering the quantum mechanical effect. Because quantum confinement and charge centroid effects are more prominent in relatively small-scale devices, these effects must be considered to model *N*_*bt*_ in the oxide layer.

We calculated the density of border traps using both physical thickness and CET while keeping other parameters constant. The *N*_*bt*_ values extracted using CET were approximately 65% lower than *N*_*bt*_ values obtained by physical thickness. The samples were also processed by different annealing treatments. FGA-annealed samples showed comparatively lower *N*_*bt*_ values than did than RTA-processed samples, although the RTA-processed samples had lower *D*_*it*_ values. The FGA treatment helps to passivate the border traps, whereas the interface traps are passivated by RTA. Moreover, samples deposited at 300 °C showed moderately low border trap concentrations because of their different stoichiometric structure, which allowed for more H_2_ incorporation during FGA. The relationship between tunneling distance and border trap concentration with respect to border trap extraction voltage was also studied. With an increase of applied voltage as the Fermi level penetrated into the conduction band, the tunneling distance was observed to be deeper from the response of more traps with relatively small electron capture areas.

## Methods

### Sample preparation and measurements

An In_0.53_Ga_0.47_As n-type heterostructure was created by metal-organic chemical vapor deposition (MOCVD) according to the procedure described here. First, two strain-relaxation buffer epitaxies of GaAs and InP were grown on a typical 300-mm n-type Si (001) substrate following the Volmer-Weber growth mode, with a carrier concentration of 2 × 10^18^ cm^−3^ on each layer and thicknesses of 350 nm and 800 nm, respectively. Then, an upper structure was formed, which consisted of a 110-nm Si-doped n-In_0.53_Ga_0.47_As layer with an electron density of 5 × 10^17^ cm^−3^ as the bottom layer and another 160-nm Si-doped n-In_0.53_Ga_0.47_As layer with an electron density of 1 × 10^17^ cm^−3^ as the upper layer.

A total of six samples of ALD Al_2_O_3_ with a configuration of 30 cycles on In_0.53_Ga_0.47_As were prepared. They were deposited at three different growth temperatures of 200 °C, 250 °C, and 300 °C, while two samples were arranged at any specific temperature. Prior to deposition of the dielectric, the In_0.53_Ga_0.47_As substrates were treated with several wet cleaning processes to remove any native oxide or other contaminants. At the start, the substrates were cleaned with acetone and isopropyl alcohol for 5 minutes each. Then, they were treated with a solution of diluted hydrochloric acid (HCl) and deionized (DI) water, maintaining a ratio of 1:10 at room temperature for 30 s to eliminate any native oxide. Subsequently, they were cleaned with deionized water for 2 minutes and dried in a nitrogen (N_2_) environment to displace the water from the wafer surface so that no water mask could form.

After the solution treatment, the substrates were transported to an ALD chamber within a minimal time interval. Before starting the actual deposition, 10 cycles of trimethylaluminium (TMA) preclean were performed because it was reported to be beneficial for passivating the interface trap density due to a self-cleaning effect^20,42,43^. Then, the Al_2_O_3_ dielectric was deposited by ALD using TMA and water (H_2_O) as the metal precursor and oxidant, respectively. The deposition process began with a TMA pulse onto the In_0.53_Ga_0.47_As surface and then was followed with a water pulse. The pulse time was 0.1 s in both cases. Between the TMA pulse and water pulse, a purge duration of 20 s was maintained. Nitrogen (N_2_) was used for both the purge and carrier gas, with a flow rate of 300 sccm. Both the TMA and water reservoirs were at room temperature.

The physical thickness (*t*_*ox*_) of the ALD-deposited Al_2_O_3_ layers was quantified by ellipsometry at an incident angle of 70°. The thicknesses were found to be 4.2006 nm, 3.867 nm, and 3.5128 nm for the 200 °C, 250 °C, and 300 °C deposition cases, respectively. Therefore, for MOS capacitor formation after Al_2_O_3_ deposition, an ALD 5-nm TiN layer was deposited on top of the dielectric. Then, a metal layer of Ti/Au (200/2000 Å) was deposited by electron-beam (E-beam) evaporation for frontside metal contact by a lift-off process. The same metal layer was again deposited for backside metal contact. Reactive-ion etching based on SF_6_/Ar Gas (30/10 sccm) was used to etch the TiN layer.

After the MOS capacitor formation, one set of samples deposited at three different temperatures was processed by RTA for 2 minutes at 350 °C in nitrogen (N_2_) ambient, whereas another set was annealed at 300 °C in forming gas (N_2_:H_2_ = 96%:4%) for 30 minutes. The C-V curves at a frequency range from 10 kHz to 1 MHz were measured at room temperature using a Keithley 4200A-SCS parameter analyzer by applying a gate voltage. The effective relative permittivity (*ε*_*r*_) and *CET* were calculated according to the following equations:

11 $${\varepsilon }_{r}=\frac{{C}_{{\rm{\max }}}{t}_{ox}}{{\varepsilon }_{0}}$$

Here, *C*_*max*_ is the maximum capacitance per unit area and *t*_*ox*_ is the oxide thickness.

12 $$CET=\frac{3.9{\varepsilon }_{0}}{{C}_{accum}}$$

Here, *C*_*accum*_ is the accumulation capacitance per unit area. The interface trap density (*D*_*it*_) was calculated through the conductance method by measuring the parallel conductance (*G*_*p*_/*ω*_*max*_)^44^.
